# Editorial: Genetics, microbiomes, and environmental factors in autoimmunity: from bench to bedside

**DOI:** 10.3389/fimmu.2026.1897679

**Published:** 2026-06-18

**Authors:** Syahrul Sazliyana Shaharir, Adli Ali, Fahd Adeeb

**Affiliations:** 1 Department of Medicine, Faculty of Medicine, National University of Malaysia, Cheras, Malaysia; 2 Department of Paediatric, Faculty of Medicine, National University of Malaysia, Kuala Lumpur, Malaysia; 3 Department of Medicine, The Royal College of Surgeons in Ireland (RCSI) and University College Dublin (UCD) Malaysia Campus (RUMC), Penang, Malaysia

**Keywords:** autoimmune, environmental, genetic, infection, inflammation

Autoimmune diseases represent a highly complex and heterogeneous group of disorders resulting from the breakdown of immune tolerance. The etiology of these conditions is increasingly recognized as a result of multifactorial interplay involving genetic susceptibility, environmental exposures such as infection and alterations in gut microbiome. This Research Topic, “Genetics, microbiomes, and environmental factors in autoimmunity: from bench to bedside,” aims to advance our understanding of the complex interactions between genetic predisposition and environmental factors in the pathogenesis and clinical aspects of autoimmune diseases. The collection of five articles presented in this editorial highlight the rapidly evolving landscape of autoimmunity, emphasizing shared genetic architectures, the impact of specific polymorphisms, and the critical role of environmental factors such as viral infections.

The global burden of autoimmune diseases is shifting, necessitating a deeper understanding of their epidemiological and genetic roots. An et al. investigated these trends by evaluating the global burden and genetic insights of Rheumatoid Arthritis (RA) and Juvenile Idiopathic Arthritis (JIA) in populations aged 0–19 years. Their analysis of the Global Burden of Disease 2021 data revealed a rising global incidence and prevalence of RA among children and adolescents over the past three decades. Utilizing two-sample Mendelian randomization analyses based on genome-wide association study (GWAS) summary statistics from European ancestry cohorts, the authors identified immune-related traits that may play causal roles in the pathogenesis of rheumatoid arthritis (RA) and juvenile idiopathic arthritis (JIA). For instance, both conditions shared risk-enhancing traits like CD28 expression on CD8+CD45RA+ T cells, CD25 expression on memory B cells, as well as circulating signaling lymphocytic activation molecule (SLAM) and Fms-like tyrosine kinase-3 ligand. These findings suggest shared pathogenic mechanisms involving T-cell co-stimulation and antigen-presentation pathways. Conversely, higher genetically predicted levels of CD4 expression on activated regulatory T cells, HLA-DR expression on dendritic cells and myeloid dendritic cells, and CD4+ expression on HLA-DR+ CD4+ T cells were associated with reduced disease risk in both RA and JIA, highlighting the potential protective role of effective antigen presentation and regulatory T-cell–mediated immune regulation. Conversely, several traits, including CD25^hi^ CD45RA− CD4+ non-Treg cells and the metabolite X-24544, demonstrated discordant associations between RA and JIA, suggesting the presence of distinct disease-specific immunometabolic pathways underlying each condition. This study underscores that immunologically informed genetic approaches could complement clinical phenotypes to refine risk stratification across the age spectrum.

Exploring the concept of shared autoimmunity across different organ systems, Jia et al. provided an integrated analysis of the genetic overlap between RA and Inflammatory Bowel Disease (IBD). While the genome-wide genetic correlation between these two distinct conditions has traditionally appeared modest, their study utilized advanced analytical tools to dissect their genetic relationship, including MiXeR, LAVA, conditional/conjunctional FDR (cond/conjFDR), PLACO, and FUMA, using large-scale GWAS datasets of European ancestry. The authors found that a profound polygenic overlap between IBD and RA. Furthermore, specific shared loci, such as TNFAIP3, COG6/TNFSF11, and JAK2, were identified and shown to be enriched in critical pathways governing T-cell activation, cytokine signaling, and Th1/Th17 differentiation. Collectively, these findings provide a more nuanced and stratified understanding of the shared genetic architecture between inflammatory bowel disease (IBD) and rheumatoid arthritis (RA), while also establishing a foundation for future mechanistic investigations and translational applications.

Individual genetic variants play a decisive role in shaping disease susceptibility and severity. García-De la Torre et al. explored the genetic underpinnings of idiopathic inflammatory myopathies (IIMs), focusing specifically on the *IL1B* rs16944 polymorphism. By analyzing a cohort of Mexican mestizo patients, they revealed a significant association between the CC and CT genotypes of this polymorphism and an increased risk of developing IIMs, particularly dermatomyositis, polymyositis, and cancer-associated myositis. The *IL1B* gene encodes interleukin-1β, a potent proinflammatory cytokine involved in cellular proliferation, differentiation, and apoptosis; thus, this genetic variation is directly implicated in the chronic inflammation and muscle tissue damage characteristic of these systemic diseases. In their cohort, enzyme levels of creatine phosphokinase (CPK) showed significant differences across genotypes, reinforcing the mechanistic link between innate immune genetics and clinical myopathy severity.

In a parallel effort to link genetic variation with clinical outcomes, Chen et al. comprehensively reviewed the genetic landscape of rheumatoid arthritis (RA), spanning susceptibility loci, disease phenotypes, and potential therapeutic targets. The authors described how highly polymorphic variants in genes such as HLA-DRB1 and PTPN22 contribute to the loss of self-tolerance and promote autoantibody production, while other genes, including CCR6 and CDK6, regulate downstream inflammatory signalling and progressive bone destruction. Importantly, they highlighted the clinical relevance of specific polymorphisms, including variants in HLA-E, NKG2D, and MICA, which may predict patient responsiveness to biologic and targeted therapies in RA. This comprehensive synthesis underscores the importance of understanding the biological and functional consequences of genetic variation in RA, not only to elucidate disease mechanisms but also to move beyond the conventional trial-and-error treatment approach. Collectively, these insights provide a strong foundation for the development of personalized diagnostics and precision therapeutic strategies tailored to individual molecular and genetic profiles.

While genetic factors establish an underlying susceptibility to autoimmunity, environmental exposures often serve as the critical triggers for disease onset. Liu et al. explored this interplay by examining the relationship between viral infections and anti-melanoma differentiation-associated gene 5 (MDA5) antibody-positive dermatomyositis (anti-MDA5+ DM), a distinct clinical subtype strongly associated with rapidly progressive interstitial lung disease (RP-ILD) and high mortality. The review highlighted distinct geographical and seasonal clustering patterns, with disease onset occurring more frequently in densely populated regions and during winter and early spring, supporting the hypothesis that respiratory viral infections and environmental exposures may act as external triggers in genetically susceptible individuals. The authors further summarized evidence implicating RNA viruses, including SARS-CoV-2 and members of the herpesvirus family, in precipitating disease onset through a proposed “two-hit” pathogenic mechanism. In this model, viral double-stranded RNA activates the cytoplasmic MDA5 sensor, leading to an exaggerated type I interferon response, while genetically susceptible individuals carrying variants such as WDFY4 exhibit enhanced innate immune activation, MDA5 overexpression, accelerated apoptosis of infected cells, and subsequent production of pathogenic anti-MDA5 autoantibodies. Collectively, this review illustrates how the interplay between environmental insults and genetic predisposition contributes to the breakdown of immune tolerance and the development of autoimmune disease.

In summary, the articles compiled in this Research Topic collectively advance our understanding of autoimmunity as a dynamic and multifactorial interplay between complex genetic architectures, immune dysregulation, and environmental triggers. From demonstrating extensive polygenic overlap between seemingly distinct diseases such as inflammatory bowel disease (IBD) and rheumatoid arthritis (RA), to identifying specific genetic polymorphisms that influence disease susceptibility, severity, and therapeutic responsiveness, these studies highlight the critical transition from bench research to bedside application. Collectively, these findings reinforce the growing importance of integrating genomics, immunology, environmental science, and multiomic technologies into autoimmune disease research. Future studies should prioritize large-scale multiethnic longitudinal cohorts, functional validation of identified genetic variants, integration of artificial intelligence and machine learning approaches, and the development of predictive biomarkers capable of guiding early diagnosis, risk stratification, and personalized therapeutic interventions.

